# Subclinical Mastitis in a European Multicenter Cohort: Prevalence, Impact on Human Milk (HM) Composition, and Association with Infant HM Intake and Growth

**DOI:** 10.3390/nu12010105

**Published:** 2019-12-30

**Authors:** Tinu Mary Samuel, Carlos Antonio De Castro, Stephane Dubascoux, Michael Affolter, Francesca Giuffrida, Claude Billeaud, Jean-Charles Picaud, Massimo Agosti, Isam Al-Jashi, Almerinda Barroso Pereira, Maria Jose Costeira, Maria Gorett Silva, Giovanna Marchini, Thameur Rakza, Kirsti Haaland, Tom Stiris, Silvia-Maria Stoicescu, Cecilia Martínez-Costa, Mireilla Vanpee, Magnus Domellöf, Eurídice Castañeda-Gutiérrez, Sagar Kiran Thakkar, Irma Silva-Zolezzi

**Affiliations:** 1 Nestlé Research, Société des Produits Nestlé SA, Route du Jorat 57, 1000 Lausanne, Switzerland; Stephane.Dubascoux@rdls.nestle.com (S.D.); michael.affolter@rdls.nestle.com (M.A.); francesca.giuffrida@rdls.nestle.com (F.G.); euridicecg@gmail.com (E.C.-G.); 2 Nestle Research, Singapore 618802, Singapore; CarlosAntonio.DeCastro@rdsg.nestle.com (C.A.D.C.); Sagar.Thakkar@rd.nestle.com (S.K.T.); Irma.SilvaZolezzi@rdsg.nestle.com (I.S.-Z.); 3 Hôpital des Enfants, CHU Pellegrin, 33000 Bordeaux, France; claude.billeaud@chu-bordeaux.fr; 4 Hospices Civils de Lyon, Neonatal intensive care unit, Hôpital Croix Rousse, 69004 Lyon, France; jean-charles.picaud@chu-lyon.fr; 5 Univ. Lyon, CarMeN Laboratory, INSERM U1060, INRA U1397, INSA Lyon, Universite Claude Bernard, 69221 Lyon 1, France; 6 Ospedale del Ponte, 21100 Varese, Italy; massimo.agosti@ospedale.varese.it; 7 Medical School, Department of Obstetrics and Gynecology, Titu Maiorescu Medicine University, 031593 Bucharest, Romania; dr.jashi@yahoo.com; 8 Hospital de São Marcos, 4700-327 Braga, Portugal; almerindabarrosopereira@gmail.com; 9 Instituto de Investigação em Ciências da Vida e Saúde, 4720-011 Braga, Portugal; costeiramj@gmail.com; 10 Hospital de S. João, 4200-319 Porto, Portugal; gorettmd@gmail.com; 11 Karolinska University Hospital, 141 86 Stockholm, Sweden; giovanna.marchini@sll.se (G.M.); mireille.vanpee@sll.se (M.V.); 12 Centre d’Investigation Clinique de Lille, Hôpital Jeanne de Flandre, 59777 Lille, France; Thameur.RAKZA@chru-lille.fr; 13 Oslo University Hospital, 0372 Oslo, Norway; UXKILA@ous-hf.no (K.H.); tom.stiris@medisin.uio.no (T.S.); 14 Polizu Hospital, 060274 Bucharest, Romania; stoicescusilvia@yahoo.com; 15 Department of Pediatrics, University of Valencia, 46010 Valencia, Spain; cecilia.martinez@uv.es; 16 Department of Clinical Sciences, Pediatrics, Umea University, 901 87 Umeå, Sweden; magnus.domellof@umu.se

**Keywords:** subclinical mastitis, European population, human milk, longitudinal study, milk macronutrients

## Abstract

**Background:** Subclinical mastitis (SCM) is an inflammatory condition of the mammary gland. We examined the effects of SCM on human milk (HM) composition, infant growth, and HM intake in a mother–infant cohort from seven European countries. **Methods:** HM samples were obtained from 305 mothers at 2, 17, 30, 60, 90, and 120 days postpartum. SCM status was assessed using HM Sodium (Na): Potassium (K) ratio >0.6. Levels of different macro- and micronutrients were analyzed in HM. **Results:** SCM prevalence in the first month of lactation was 35.4%. Mean gestational age at delivery was lower and birth by C-section higher in SCM mothers (*p* ≤ 0.001). HM concentrations of lactose, DHA, linolenic acid, calcium, and phosphorous (*p* < 0.05 for all) was lower, while total protein, alpha-lactalbumin, lactoferrin, albumin, arachidonic acid to DHA ratio, *n*-6 to *n*-3 ratio and minerals (iron, selenium, manganese, zinc, and copper) were higher (*p* < 0.001 for all) in mothers with SCM. There were no differences in infant growth and HM intake between non-SCM and SCM groups. **Conclusion:** We document, for the first time, in a large European standardized and longitudinal study, a high prevalence of SCM in early lactation and demonstrate that SCM is associated with significant changes in the macro- and micronutrient composition of HM. Future studies exploring the relation of SCM with breastfeeding behaviors and developmental outcomes are warranted.

## 1. Introduction

Subclinical mastitis (SCM) is an asymptomatic, inflammatory condition of the lactating mammary gland [1] associated with early lactation failure [2] and poor infant weight gain [1,3]. Studies among different ethnicities have reported varying estimates of SCM prevalence reaching to as high as 66% with most of the cases occurring in early lactation and typically declining over time [1,4,5]. Manifestation of SCM is seen as a mammary gland inflammation often due to milk stasis [6], infection [7], and/or micronutrient deficiency [8]. Occasionally, SCM may progress to clinical mastitis, presenting with symptoms such as fever, mammary gland pain and reddening, combined with breast engorgement [9,10,11]. Clinical and subclinical mastitis share certain unique clinical features that include unilateral orientation, and elevated milk sodium and interleukin-8 (IL-8) concentration, which also serve as the diagnostic markers for identifying these conditions [12]. The pathophysiological changes occurring in the inflammatory mammary gland of SCM mothers comprise involuntary opening of the tight junctions around alveolar cells due to transient release of cytokines, damaging the mammary epithelial layer with resultant increase in permeability [1]. The net outcome of this enhanced permeability is increased sodium and decreased potassium concentration in the Human Milk (HM) which routinely forms the basis for employing an elevated sodium: Potassium (Na:K) ratio as a diagnostic tool for SCM in humans [12,13]. This is also supported by the studies reporting more immune cells, increased sodium, chloride, immunoglobulins [14,15], and decreased concentration of lactose [14,15,16], calcium, phosphorus, and potassium in the bovine milk of SCM cows [14,17]. In addition, Na:K ratio is reported to be a better indicator of SCM due to higher precision and accuracy in comparison to other diagnostic markers [17].

Data on SCM among humans are scarce and inconsistent. The lack of consistency may be attributed to the fact that most of our knowledge is extrapolated from preclinical studies [18,19,20,21,22], and the limited number of clinical observations offer several limitations. Most clinical studies are focused on the elevated cytokines or changes in inflammatory mediators occurring in the HM of SCM mothers in the absence of data about milk intake and/or infants’ growth [8,17,23]. However, study on milk intake by infants of SCM mothers failed to take into account the compositional changes occurring in the HM [24]. Lastly, numerous studies on HIV-infected SCM mothers reported elevated HIV viral load in milk of SCM mothers, increasing risk of HIV transmission to the newborns [3,5,12,13,25].

We conducted a prospective, longitudinal study in a large European cohort to determine the association of SCM with the composition of HM with a secondary interest to compare the milk intake and growth of infants born to SCM versus non-SCM mothers. To the best of our knowledge, this is the first report among European lactating mothers describing altered composition of several macro- and micronutrients in the HM of mothers with SCM.

## 2. Materials and Methods

### 2.1. Study Design and Population

The data presented in this longitudinal, observational study named ATLAS were collected at 13 centers across seven European countries between December 2012 and January 2016. The study centers were selected in order to have a geographical spread that could potentially reflect the variability in the HM composition (centers from Sweden and Norway representing the Nordic countries; centers from Italy Portugal, and Spain representing Western and Southern Europe; centers from France representing central Europe; and centers from Romania representing Eastern Europe). Each of the study sites was evaluated for site feasibility and these were finalized based on availability of resources at facilities. The study was approved by the institutional and local ethical boards for each center and was registered at ClincalTrials.gov with identifier NCT01894893. A written informed consent was provided by all mothers in their respective local languages.

Pregnant women were screened at clinical sites for the fulfillment of the following inclusion criteria: Aged between 18 to 40 years, especially in last trimester; body mass index (BMI) between 19 and 29, both inclusive; intend to breastfeed for at least four months; and willing to comply with the study protocol. Women who did not meet any inclusion criteria or presented with condition or on medication that contraindicate breastfeeding were excluded from the study. The mother–infant pairs enrolled into this study were observed during regularly scheduled visits carried out at Day 2, 17, 30, 60, 90, and 120 postpartum. This study was designed to investigate the impact of SCM (diagnosed as Na:K ratio in HM >0.6) on milk composition and the association between SCM, HM-intake and infant growth.

### 2.2. Maternal and Infant Characteristics

Maternal and infant data were collected by trained and certified research nurses and assistants. Maternal characteristics included demography, anthropometry, medical history, and medication use. Infant characteristics included anthropometry, infant intake diary (three centers in France), and medication use.

### 2.3. Collection of HM Samples and Diagnosis of SCM

A standardized procedure was employed to collect milk samples from the same breast throughout the study period using an electric breast pump (Medela Symphony, Switzerland) at a scheduled time of 11:00 ± 2:00 h to avoid circadian influence. To achieve full milk extraction of the single breast, all enrolled mothers were asked to empty the breast in the previous feed. During the scheduled visit, the selected breast was emptied completely, and an aliquot of 40 mL was reserved for further analysis. The rest was returned to the mother for feeding the infant. Each sample was collected in a labeled freezing tube and stored at −18 °C until it was delivered to the Nestlé Research Centre (Lausanne, Switzerland) where it was stored at −80 °C for further analysis.

SCM status was assessed using HM sodium potassium ratios (Na:K). Lactating mothers were categorized in to two groups: those having SCM (defined as Na:K ratio >0.6) versus normal (defined as Na:K ratio ≤0.6) based on the Na:K ratios in HM in early lactation (days 2, 17, and 30) [17]. Lactating mothers having at least one incidence of SCM during any of these three time points were classified as SCM, while those in the normal category did not have any incidence of SCM in any of these time points. The cut-offs are based on the published literature on human SCM [12,13,17].

### 2.4. Macronutrient Composition of HM

Levels of lactose, lipid, and energy were measured in HM samples employing HM analyzer generation 3 (MIRIS AB, Uppsala, Sweden) supported by XMA-SW software version 2.87, that works on the principle of semisolid middle infrared (MIR) transmission spectroscopy. All samples were homogenized for 3 × 10 s using a sonicator (MIRIS AB, Uppsala, Sweden) and were kept in a water bath at 40 °C prior to measurement. An in-house control sample was analyzed after every tenth measurement for quality control purposes.

Fatty acid (FA) profile—18:3 N-3-Octadecatrienoic Acid (linolenic acid), 22:6 N-3-Docosahexanoic Acid (DHA), arachidonic acid to DHA ratio (ARA/DHA ratio), *n*-6 to *n*-3 ratio—was determined by the procedure of Cruz-Hernandez et al. [26]. The analysis of methyl esters of FA (FAMEs) was performed by gas chromatography using a CP-Sil 88 capillary column (100 m, 0.25 mm, id. 0.25 µm film thickness) and their identification was done by comparing the retention time with authentic standards (GC standard Nestlé 36 from NuCheck-Prep, Elysan, MN, USA).

Total protein content in HM was measured using the colorimetric bicinchoninic acid (BCA) method according to the manufacturer’s protocol of BCA assay kit (ThermoFisher Scientific, Waltham, MA, United States). The four major HM proteins: alpha-lactalbumin, lactoferrin, serum albumin, and caseins were quantified using a LabChip system [27].

### 2.5. Mineral and Trace Elements Concentration of HM

Quantification of minerals—Sodium (Na), Magnesium (Mg), Phosphorous (P), Potassium (K), Calcium (Ca), Manganese (Mn), Iron (Fe), Copper (Cu), Zinc (Zn) and Selenium (Se)—were carried out using an internal methodology based on inductively coupled plasma mass spectrometry (ICP-MS), PerkinElmer model NexION 300. HM sample (0.7 mL) was transferred into perfluoroalkoxy alkane vessels and mineralized in a CEM^®^ Microwave digestion system using HNO_3_/H_2_O_2_. Mineralized samples were transferred to polyethylene tubes, diluted with Milli-Q water. Germanium (Ge) and Tellurium (Te) were added as internal standards. Quantification was realized by ICP-MS using He or CH_4_ as collision or reaction gas. Certified Reference Materials were added to all analytical series to control the quality of the quantification.

### 2.6. Estimation of Milk Intake by Test Weighing Technique

A modified test weighing method was employed to determine the HM intake in a subset of the infants [28]. The procedure involved mothers test weighing their infants using an electronic balance (Seca 354) before and after the selected feeding sessions for three times on a single day as close as possible to an upcoming study visit. All enrolled mothers recorded the infant weight and number of feeds in the diaries provided by the study center and the calculations were made by the study staff to estimate the milk intake per feed. Daily milk intake was estimated by taking the average of three feeds and multiplying by the total number of feeds on that day.

### 2.7. Infant Growth Parameters

All growth parameters were assessed using standard procedure by trained personnel. Infants were weighed without clothing or diaper in the same electronic scale (Seca 354) throughout the study period. Length was measured in the recumbent position using a standardized length board taking care of complete alignment and full body extension with flexed feet. Head circumference was measured by a standard non-elastic plastic-covered measurement tape to the nearest 1 mm.

### 2.8. Statistical Analysis

All statistical analyses were done using R version 3.2.3. The descriptive statistics for continuous variables are presented with number (n) of subjects, mean and standard deviation; for categorical data number (n) with percentage (%) are presented.

For comparison of HM composition between mothers with SCM versus normal, a linear mixed model was applied explaining the milk concentration (of a particular nutrient) with the age of infant, SCM status (including the interaction between the age and the SCM status), the country of origin, the mode of delivery, and the gestational age of the infant at birth. Global estimates were calculated using analysis of variance on the model to identify variables significantly influencing the milk concentration.

For comparison of infant growth parameters between mothers with SCM and normal, a linear mixed model was applied explaining the infant’s growth (Z-scores: weight for age, weight for length, length for age, BMI for age and head circumference for age) with visit, SCM status (including the interaction between visit and the SCM status), the baseline value of the corresponding growth parameter (value at first visit), and the gestational age of the infant at birth. The WHO growth charts were used for calculation of Z-scores.

For comparison of infant milk intake between mothers with SCM and normal, a linear mixed model was applied explaining the milk intake with visit, SCM status (including the interaction between visit and the SCM status), the breastfeeding compliance of infant, and the weight of infant per visit. This exercise was performed for daily milk intake, average milk intake per feeding, as well as number of feeds per day.

Contrast estimates were calculated per visit by comparing mothers with and without SCM. Statistical significance is considered when *p*-values are <0.05.

## 3. Results

### 3.1. Study Population

**Figure 1:**
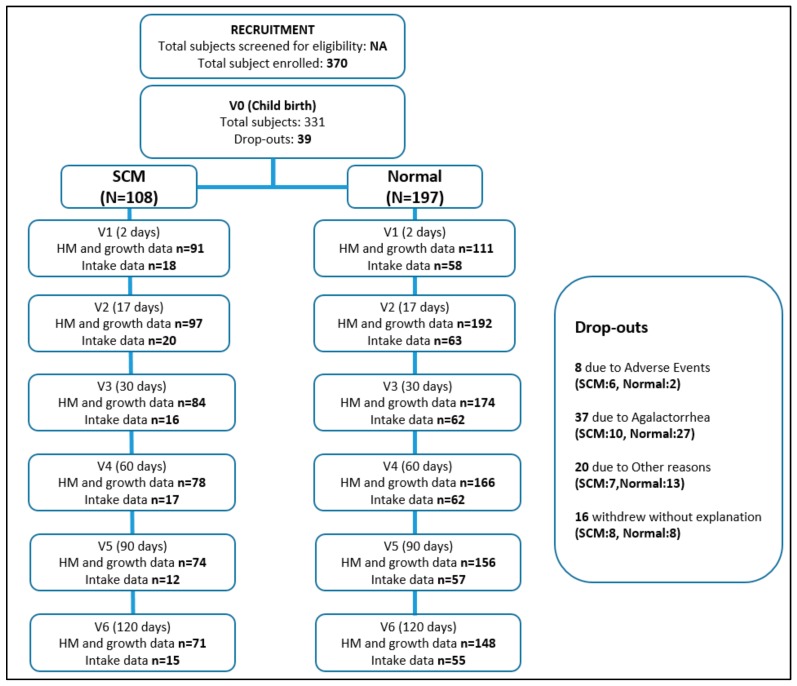
Flowchart outlining number of mothers per group at each visit. The Subclinical mastitis (SCM) group in this flowchart indicates mothers with at least one incident of SCM in the first month.

A total of 331 out of 370 screened pregnant women from seven European countries reached to baseline for further analysis. Detailed description about number of women at all six visits is outlined in Figure 1.

### 3.2. SCM Prevalence

**Figure 2:**
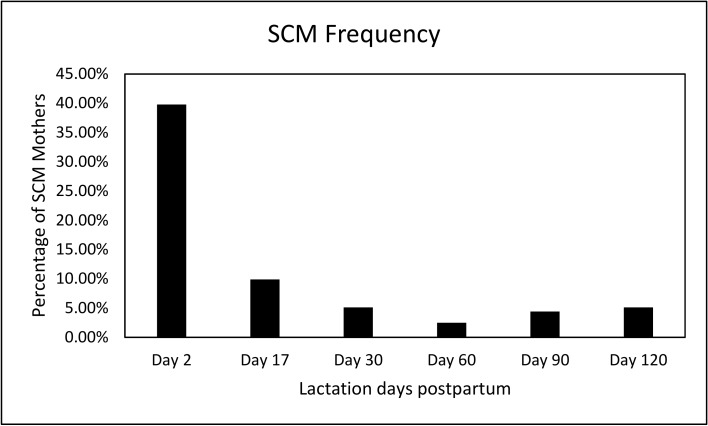
Frequency of SCM in a European cohort of lactating mothers across four months of lactation.

Figure 2 shows the percentage of mothers with SCM across the first four months of lactation, with the highest prevalence reported at day 2 (39.6%) and thereafter decreasing over the four-month period (9.8% at day 17, 5% at day 30, 2.5% at day 60, 4.4% at day 90, and 5% at day 120). As our definition of SCM stated previously (at least one incidence of SCM during day 2, 17, and 30), the proportion of mothers having SCM was 35.4% (108 out of 305 mothers). There were no significant differences between the proportions of dropouts in SCM vs. normal mothers.

### 3.3. Maternal and Infant Characteristics

Maternal and infant characteristics of both (SCM and normal) groups adjusted for visit, country, delivery mode, and gestational age are shown in Table 1. A total of 108 lactating mothers participating from different European countries were diagnosed with SCM. The anthropometric indicators like maternal age, height, pre-pregnancy weight, BMI, and parity were not significantly different between the SCM and normal groups. However, there were significant country-wise differences in the frequency of SCM (*p* < 0.001). Notably the gestational age at delivery was significantly lower among mothers with SCM compared to normal (*p* < 0.001). Similarly, the rates of caesarean mode of delivery were higher among mothers with SCM (*p* < 0.001) and the infant birth weights were also significantly low (*p* = 0.001) compared to their normal counterparts.

### 3.4. Macronutrient Composition of HM

Essential macronutrients were examined in the milk of SCM and normal mothers as shown in Table 2. While there were no differences in the levels of energy and fat in the milk from mothers of both groups, the levels of lactose were significantly lower in the milk from SCM mothers (based on overall comparison across the six visits) (*p* < 0.001), with specific time point differences observed at day 2, 17, and 30 (*p* < 0.05 for all). The HM concentration of total protein was also significantly higher in SCM mothers compared to normal mothers (based on overall comparison across the six visits) (*p* < 0.0001), with specific time point difference only at day 2 (*p* < 0.001).

**Figure 3:**
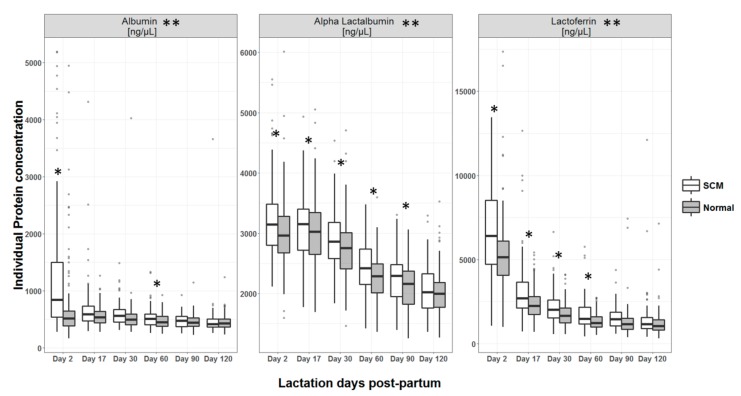
Concentrations of individual proteins in HM across lactation by presence or absence of SCM. * Statistical significance of the difference between mothers with SCM and without SCM for a particular time point with *p* value <0.05, ** *p* value for overall difference between groups <0.001.

Quantification of four major HM proteins revealed that the levels of alpha-lactalbumin, lactoferrin, and albumin in milk of SCM mothers were higher when compared to normal (based on overall comparison across the six visits) (*p* < 0.001 for all) (Figure 3). More specifically, for alpha-lactalbumin significant differences were observed at day 2 and 60; for lactoferrin at day 2, 17, 30, 60 and 90; and for albumin at day 2, 17, 30, 60 (*p* < 0.05 for all). Total caseins were not significantly different between the two groups.

**Figure 4:**
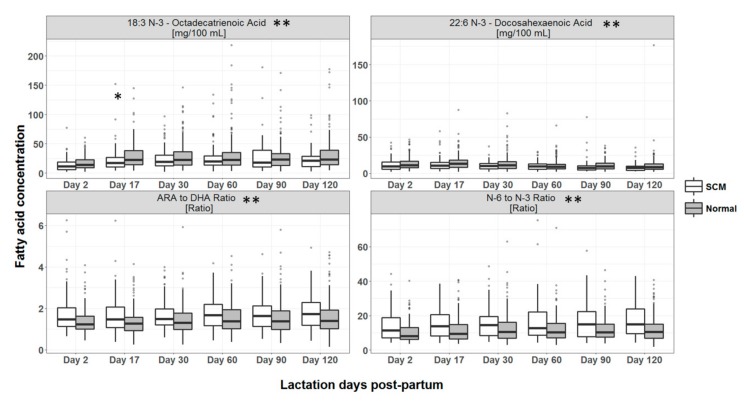
Concentrations of fatty acids in HM across lactation by presence or absence of SCM. * Statistical significance of the difference between mothers with SCM and without SCM for a particular time point with *p* value <0.05, ** *p* value for overall difference between groups <0.001.

The fatty acid profile of HM from SCM mothers contained lower 22:6 (n-3) docosahexanoic acid (DHA) and 18:3 (n-3) octadecatrienoic acid (linolenic acid), while levels of arachidonic acid to DHA ratio, and *n*-6 to *n*-3 ratio were higher than the normal group (based on overall comparison across the six visits) (*p* < 0.001 for all) (Figure 4). More specifically, per time point differences were seen only for 18:3 (n-3) octadecatrienoic acid at day 17 (*p* < 0.05).

### 3.5. Mineral and Trace Element Concentration in HM

Milk of SCM mothers had significantly lower calcium (*p* = 0.003) and phosphorus (*p* < 0.0001), while iron, selenium, manganese, zinc (*p* < 0.0001 for all), and copper (*p* < 0.001) were significantly higher in the HM of SCM mothers compared to their adjusted normal group (based on overall comparison across the six visits, Table 3). Considering per time point, significant differences were observed for calcium, selenium, copper, manganese, and zinc at day 2 (*p* < 0.05), iron at day 2 and 17 (*p* < 0.05 for both), and for phosphorus at day 2, 17, and 30 (*p* < 0.05 for all).

### 3.6. SCM Status and HM Intake

Feeds per day (feeding frequency) and HM intake by test weighing were recorded and daily intake estimated for a subset of infants from three French sites between the two groups, as described in Table 4.

The median (Q1, Q3) per feed intake (kg) of milk by infants at each time point (day 2, 17, 30, 60, 90, and 120) was 0.026 (0.016, 0.04), 0.07 (0.04, 0.09), 0.09 (0.06, 0.11), 0.1 (0.07, 0.14), 0.12 (0.09, 0.16), 0.13 (0.09, 0.16), respectively. Similarly, the median daily intake (kg) of HM by infants at each time point (day 2 till day 120) was 0.27 (0.17, 0.36), 0.6 (0.43, 0.72), 0.68 (0.54, 0.84), 0.72 (0.60, 0.88), 0.76 (0.62, 0.96), 0.75 (0.66, 0.88), respectively (data not shown). There was no difference in feeding frequencies and per feed intake by infants of SCM and normal mothers. The statistical modeling without covariates also showed no differences between groups at any time points except at day 90, where a slightly higher median was observed for SCM group. We further incorporated infant weight and breastfeeding behavior (exclusively, partially, or predominantly) as covariates in the model and observed that among a partially breastfed group of SCM mothers, infants consumed significantly (*p* = 0.032) higher daily intake of milk compared to their non-SCM counterparts at day 90. However, this was not observed in the exclusively and predominantly breastfed group suggesting a possibility of compensatory behavior by infants for overall milk intake amongst the partially breastfed subset.

### 3.7. Infant Growth Parameters

Infants born to mothers with SCM had smaller head circumference (*p* < 0.0001), weight (*p* = 0.0005), weight for length (*p* = 0.004), and BMI (*p* = 0.0005) at birth, in addition to having a lower gestational age at birth, as shown in Table 5. However, the differences disappeared as lactation progressed and anthropometric measurements were not statistically significant between groups across the study.

## 4. Discussion

This study reported 35.4% SCM prevalence during the first month of lactation among European mothers using a diagnostic marker of HM Na:K ratio >0.6. The great majority of cases were in the first two days of lactation, ranging from 40% at day 2 to 10% at day 17. The prevalence of SCM in our cohort decreased with the progression of lactation (two months and later). These observations are in agreement with a recent study on Guatemalan mothers that reported SCM prevalence of 30% between 5 and 17 days of lactation which reduced to 15.6% between 18 and 46 days of lactation [17]. There are wide variations in the estimate of SCM prevalence from data among different ethnicities [1,4,5,8]. This variation may be attributed to the lactation stage taken into consideration or differences in SCM definitions that include milk Na:K >1.0 [4] or milk Na:K >0.6 to ≤1 [4] or milk leukocyte count >1 million cells/mL [5].

### 4.1. Maternal and Infant Characteristics

In our dataset we observe that normal mothers and those with SCM are different in terms of country representation. Notable differences are Romania (26% of SCM group while only 7% of the normal group) and France (20% for SCM group while 34% in normal group). Romania had the highest incidence rate of SCM at 67% (total number of mothers = 42) while the lowest incidence rate can be found in Norway with 0 incidents of SCM (total number of mothers = 10). This significantly different distribution of SCM in the European countries may be indirectly associated with the differences in the breastfeeding practices and/or the mode of delivery being practiced in these countries as reported by an Euro-Peristat study that also mentions a higher proportion of C-sections being followed in countries such as Portugal and Romania [29]. Previous literature also shows that women undergoing C-sections have a greater likelihood of developing mastitis [30]. We recorded a similar observation of higher SCM incidence in mothers who underwent caesarean mode of delivery (*p* < 0.001). This C-section associated SCM may be attributed to the delay in breastfeeding consequently leading to breast engorgement, milk stasis, and inadequate breast emptying [9]. There was no significant difference in the maternal age, height, pre-pregnancy weight, BMI, parity, infant’s length, and gender between the SCM and normal groups. However, mothers with SCM had significantly shorter gestational age at delivery and their infants were of lower weight at birth when compared to normal mothers.

### 4.2. Macronutrient Composition of HM

To the best of our knowledge, this is the first report to demonstrate the altered levels of macronutrients in the HM of mothers with SCM versus those without in a cohort of European population, although there are a few similar observations from mothers with mastitis of different ethnicities [31,32].

In HM, lactose is the main sugar that works as an osmotic regulator [31]. We observed significantly lower lactose concentrations in the HM of SCM mothers. This observation is corroborated by the studies of Prentice et al. and Fetherston et al. that reported a similar finding [33,34]. Diminished lactose levels in the milk of mothers with breast infection is also reported by the study of Ramadan and coworkers [35]. This decreased lactose in the milk of mothers with mastitis may be attributed to its leaking into the bloodstream, whereas in the infected HM reduced biosynthesis is postulated as the possible mechanism for low lactose [33,34,35]. Depending on the magnitude of SCM condition, one or both might be the possible mechanism(s) to reduce lactose in milk of SCM mothers. In our cohort, we did not observe any differences in the energy values of HM between mothers with SCM and those without. To the contrary, Say and coworkers observed lower HM energy levels in mastitic mothers from Turkey in a case-control study. The authors postulated this effect as a result of decreased milk synthesis, enhanced permeability of blood–milk barrier and/or increased proteolytic/enzymatic activity [31].

Our study reported higher levels of total protein and specific proteins in the milk of mothers with SCM. Most of the information available today either comes from bovine studies or from comparing lactating mothers with and without mastitis, and results are conflicting. In dairy bovine herds experiencing intramammary infection, the non-casein nitrogen fraction was found to be elevated, while the casein content and casein-to-total protein were lower compared to normal quarters. The authors postulated this to be due to increased proteolysis resulting from the elevation of plasmin or other proteases derived from somatic cells, leading to the breakdown of casein and the influx of blood proteins that are relatively resistant to proteolysis (e.g., immunoglobulins, IgG, and bovine serum albumin) into milk via the paracellular pathways that proliferate during mastitis [36]. Similar observations were also made in seasonally calving dairy herds where milk from cows with mastitis had higher concentrations of total protein, non-casein proteins, but lower concentrations of casein proteins compared to milk from healthy cows [37]. No significant differences were observed in the HM protein levels between Turkish mothers with and without mastitis, and the authors attributed this to ethnic differences, differences in maternal nutritional status and dietary intake, breastfeeding patterns and differences in milk measurement techniques [31]. The evidence with respect to specific proteins such as lactoferrin is also conflicting where in some studies it is shown to be increased in mothers with mastitis [34] while in others it does not demonstrate an increase, despite an increase in other milk components such as IL-1ß and IL-6 [38]. The observed higher levels of proteins in mothers with SCM may be related to the opening of the tight junctions and the influx of plasma proteins into milk that may have exceeded the enzymatic breakdown of proteins synthesized de novo resulting in a net increase in total protein. Typically, proteins such as alpha lactalbumin have components such as bioactive peptides that possess antibacterial properties [39].

Fat is the most variable macronutrient present in breast milk [31]. Also, reported data offer intriguing evidences of lipolysis due to inflammation [31]. Consequently, a number of studies have reported low fat concentration in the milk of mastitic mothers attributed to the given mechanism or reduced synthetic or secretory capacity of the mammary gland [31,37]. However, in our data set, we did not observe any differences in the HM fat between the two groups.

Ours is the first study to report that SCM is associated with alteration of the levels and ratios of HM fatty acids. The milk of mothers with SCM had lower levels of 22:6 *n*-3-docosahexanoic acid (DHA) and 18:3 *n*-3 –octadecatrienoic acid (linolenic acid), while higher arachidonic acid to DHA ratio, and *n*-6 to *n*-3 ratio compared to normal mothers. This observation points towards a pro-inflammatory state of SCM, whereby the low milk DHA levels in SCM mothers might be associated to its uptake for attenuating inflammation, since it is a known precursor of resolvins (compounds that resolve inflammation) [40].

### 4.3. Mineral and Trace Element Concentration in HM

Our data shows higher levels of iron, selenium, manganese, zinc, and copper in the milk of mothers with SCM and lower levels of calcium and phosphorus. The role of selenium in preventing mastitis has been well documented in bovine studies. Deficiency of selenium is associated with reduced and impaired polymorphonuclear neutrophils activity (PMN), thereby influencing cell phagocytic capacity, bactericidal activity or both [41]. Dietary supplementation of cows with selenium and vitamin E results in a more rapid PMN influx into milk following intramammary bacterial challenge and increased intracellular killing of ingested bacteria by PMN, as well as lowering the frequency and shortening the duration of clinical mastitis [42,43]. Selenium is known to exhibit anti-inflammatory effects and can attenuate inflammation possibly by inhibiting the activation of NF-κB by modulating selenoprotein genes expression [44] The only other study in humans that has investigated the impact of SCM on milk mineral concentrations was a cross-sectional study among lactating mothers from Guatemala. HM samples were obtained during transitional lactation (5–17 days), early lactation (18–46 days) and established lactation (4–6 months) and mineral concentrations were analyzed using the similar methodology as ours. Interestingly, they also found that SCM was associated with higher levels of selenium and lower levels of phosphorus in the HM, even after controlling for the lactation stage. The authors concluded that the accumulation of selenium in the HM of SCM mothers may be an indirect consequence of hyper-accumulation of selenium at the inflamed site and may be an important defense mechanism for the mammary gland against SCM pathogens [17]. Similar to selenium, we also observed for the first time higher concentrations of iron, manganese, zinc, and copper in the milk of mothers with SCM and this may be again a mechanism to combat inflammation. Previously animal studies have shown that supplementation with copper and zinc reduces the somatic cell count in milk and enhances the immune system [45,46]. Deficiencies in minerals such as selenium, copper, and zinc can lead to prolonged inflammation, increased accumulation of reactive oxygen species, reduced immune cell proliferation and activity, as well as diminished ability for intracellular killing of mastitis pathogens. Reports from studies in cows with mastitis also indicate a decrease in milk calcium concentrations of up to 11% [47]. Additionally, lower serum levels of calcium have been associated with impaired phagocytosis and with lower neutrophil activation in cows [48] The lower concentrations of phosphorus observed in the HM of mothers with SCM might be related to its increased incorporation into Adenosine triphosphate for energy production due to inflammation.

### 4.4. SCM Status and HM Intake

Evaluation of HM intake is often done in clinical as well as research settings for various purposes. The double label water technique is usually referred to as a reference method but is resource intensive and bears a heavy subject burden. Alternative methods such as test weighing a breastfed infant before and after feeding can provide a good estimate using a sensitive balance [28,49,50,51]. Even test weighing is reported in literature with different frequency throughout the day. This can vary from weighing the infant at each feeding for 24 h [49] or 12 h [24] and in our study where we weighed the infants on three occasions to calculate an average by recording the number of feeds per day. Even with this limitation in our test weighing methodology our findings suggested that there were no differences in number of feeds per day and per feed milk intake by infant between the groups of mothers with SCM or no SCM. This observation is in agreement with Aryeetey et al., where infants were weighed for 12 h per day [24]. In contrast, Manganaro et al. have reported an inverse relationship between HM sodium and infant milk intake, however, that study was limited to only first week postpartum [52]. Overall this may suggest that SCM may have an impact on infant milk intake but the effect is limited to the first week only and has no to minimal impact at later stages of lactation as demonstrated in our study.

### 4.5. Infant Growth Parameters

A recent study on Guatemalan mothers exhibited low birth weight (lower WAZ score) and head circumference in infants born to mothers who later developed SCM (Na:K > 0.6) as compared to normal [53]. This observation is in agreement with our study wherein infants of mothers who developed SCM were found to be lighter with a smaller head circumference which may be explained due to greater incidence of shorter gestational age and higher rates of C-section. A similar observation is also reported by Say et al. but on mastitic mothers [31].

After one month of lactation the incidence of SCM decreased drastically, the differences in infant weight and head circumference also disappeared and were not significant over the study period. Other studies have reported that subclinical mastitis causes poor infant weight gain [1,3,25,54]. These studies were performed in African populations, and in some of them the incidence of HIV is high. In a recent study among Mam-Mayan mothers at both early (2–46 d) and established (4–6 mo) lactation, SCM was inversely associated with the weight-for-length Z-scores [55]. Since our study was performed in well-nourished healthy European women, we speculate that this could have contributed to better growth recovery on the infants of SCM mothers.

## 5. Conclusions

The strengths of our study include standardized and longitudinal HM collection, use of an accepted diagnostic marker of SCM such as the ratios of Na and K in HM, detailed characterization of several constituents of HM including energy, macronutrients, fatty acids, minerals, and an assessment of infant growth over four months of lactation. As limitations, HM intake data was available only in a subset of infants from France, breastfeeding and infant feeding behaviors were not captured, no clinical data were available on mastitis or SCM, the study was not powered to perform country-wise comparisons due to very small numbers in some countries and no formal sample size calculations were done since the study was exploratory in nature.

In conclusion, for the first time, in the largest European standardized and longitudinal milk collection study among lactating mothers, we demonstrate a high prevalence of SCM in early lactation using a diagnostic marker of the ratio of Na and K in HM. The prevalence of SCM varies remarkably across countries, suggestive of differences in breastfeeding practices and/or the frequencies of C-section in these countries. SCM is significantly associated with alterations in the macro- and micronutrient composition of HM; we hypothesize these alterations may be due to the opening of the tight junctions and increased permeability of the mammary epithelium, acting potentially as a mechanism to combat inflammation. Future studies should focus on understanding the relation of this diagnostic marker of SCM with breastfeeding behaviors, HM output of the mother, infant growth and developmental outcomes.

## Figures and Tables

**Table 1:** Maternal and infant characteristics by presence or absence of SCM.

	SCM (N = 108), n (%)	Normal (N = 197), n (%)	*p* Value
***Maternal Characteristics***			
Age (y) ^a^	31.44 ± 4.42	31.17 ± 4.09	0.596
Country of residence ^b^			
Spain (56% SCM prevalence)	5 (4.8)	4 (2.0)	<0.001
France (24% SCM prevalence)	21 (20.2)	67 (34.0)	
Italy (7% SCM prevalence)	1 (1.0)	14 (7.1)	
Norway (0% SCM prevalence)	0 (0.0)	10 (5.1)	
Portugal (37% SCM prevalence)	36 (33.3)	62 (31.5)	
Romania (67% SCM prevalence)	28 (25.9)	14 (7.1)	
Sweden (40% SCM prevalence)	17 (15.7)	26 (13.2)	
Height (cm) ^a^	164.86 ± 6.06	164.89 ± 5.99	0.964
Pre-pregnancy weight (kg) ^a^	61.79 ± 8.18	61.63 ± 7.49	0.866
Pre-pregnancy BMI (kg/m^2^) ^a^	22.73 ± 2.78	22.67 ± 2.52	0.847
BMI category ^b^			
Normal weight	85 (78.7)	158 (80.2)	0.871
Overweight	23 (21.3)	39 (19.8)	
Parity ^b^			0.503
Primiparous	84 (77.8)	141 (71.6)	
Multiparous	24 (22.2)	56 (28.4)	
Gestational age at delivery (wk) ^a^	39.07 ± 1.23	39.56 ± 1.13	0.001
Mode of delivery ^b^			
Caesarian	41 (38.0)	35 (17.8)	<0.001
Vaginal	67 (62.0)	162 (82.2)	
***Infant Characteristics***			
Infant weight at birth (kg) ^a^	3.23 (0.51)	3.42 (0.44)	0.001
Infant length at birth (cm) ^a^	49.69 (2.21)	50.13 (1.97)	0.086
Infant gender ^b^			
Female	46 (42.6)	93 (47.2)	0.513
Male	62 (57.4)	104 (52.8)	

^a^ Continuous variables are presented by mean ± SD and analyzed using *t*-test. ^b^ Categorical variables are presented by n (%) and analyzed chi-square/Fisher’s exact test.

**Table 2:** Composition of Human Milk (HM) across four months of lactation by presence or absence of SCM.

Mean ± SD		Day 2	Day 17	Day 30	Day 60	Day 90	Day 120	Overall *p* Value ^1^
**Energy (kcal/100 mL)**	SCM	60 ± 10	69 ± 10	71 ± 14	72 ± 13	70 ± 15	70 ± 14	0.13
		(*n* = 36)	(*n* = 91)	(*n* = 80)	(*n* = 77)	(*n* = 70)	(*n* = 68)	
	Normal	62 ± 9	73 ± 12	72 ± 13	72 ± 15	71 ± 16	72 ± 17	
		(*n* = 55)	(*n* = 172)	(*n* = 156)	(*n* = 153)	(*n* = 150)	(*n* = 136)	
**Lactose (g/100 mL)**	SCM	6.7 ± 0.5 *	7.2 ± 0.4 *	7.3 ± 0.4 *	7.2 ± 0.4	7.3 ± 0.3	7.3 ± 0.3	<0.001
		(*n* = 36)	(*n* = 91)	(*n* = 80)	(*n* = 78)	(*n* = 71)	(*n* = 68)	
	Normal	7.0 ± 0.3	7.3 ± 0.3	7.4 ± 0.3	7.3 ± 0.3	7.3 ± 0.3	7.3 ± 0.3	
		(*n* = 55)	(*n* = 172)	(*n* = 157)	(*n* = 153)	(*n* = 150)	(*n* = 139)	
**Total protein (g/100 mL)**	SCM	2.7 ± 1.1 **	1.7 ± 0.4	1.5 ± 0.3	1.4 ± 0.3	1.3 ± 0.3	1.3 ± 0.3	<0.0001
		(*n* = 104)	(*n* = 104)	(*n* = 91)	(*n* = 80)	(*n* = 77)	(*n* = 77)	
	Normal	2.5 ± 1.1	1.6 ± 0.3	1.4 ± 0.3	1.3 ± 0.3	1.3 ± 0.3	1.3 ± 0.5	
		(*n* = 150)	(*n* = 188)	(*n* = 171)	(*n* = 162)	(*n* = 155)	(*n* = 147)	
**Total fat (g/100 mL)**	SCM	2.6 ± 1.0	3.7 ± 1.1	4.0 ± 1.5	4.2 ± 1.5	4.0 ± 1.7	4.0 ± 1.5	0.11
		(*n* = 41)	(*n* = 92)	(*n* = 80)	(*n* = 78)	(*n* = 71)	(*n* = 68)	
	Normal	2.9 ± 1.0	4.2 ± 1.4	4.1 ± 1.4	4.1 ± 1.6	4.0 ± 1.8	4.2 ± 1.8	
		(*n* = 55)	(*n* = 173)	(*n* = 156)	(*n* = 153)	(*n* = 150)	(*n* = 136)	

^1^ The macronutrient composition of HM across lactation was analyzed by using linear mixed model with milk concentration (of a particular nutrient) as dependent variable with the age of infant, SCM status, interaction between the age and the SCM status, the country of origin, the mode of delivery, and the gestational age of the infant at birth as explanatory variables. * Statistical significance of the difference between mothers with SCM and without SCM for a particular time point with *p* value <0.05, ** with *p* value <0.001.

**Table 3:** Mineral and trace element concentrations of HM across four months of lactation by presence or absence of SCM.

		Day 2	Day 17	Day 30	Day 60	Day 90	Day 120	Overall *p* Value ^1^
**Calcium (mg/L)**	SCM	256 ± 73 *	281 ± 54	296 ± 47	299 ± 41	288 ± 45	271 ± 36	0.003
		(*n* = 91)	(*n* = 97)	(*n* = 84)	(*n* = 78)	(*n* = 74)	(*n* = 71)	
	Normal	295 ± 72	293 ± 55	296 ± 51	301 ± 45	297 ± 48	284 ± 42	
		(*n* = 111)	(*n* = 192)	(*n* = 174)	(*n* = 166)	(*n* = 156)	(*n* = 148)	
**Phosphorus (mg/L)**	SCM	109 ± 41 *	150 ± 31 *	149 ± 29 *	138 ± 21	128 ± 18	126 ± 20	<0.0001
		(*n* = 91)	(*n* = 97)	(*n* = 84)	(*n* = 78)	(*n* = 74)	(*n* = 71)	
	Normal	144 ± 32	169 ± 28	155 ± 24	139 ± 21	133 ± 20	131 ± 22	
		(*n* = 111)	(*n* = 192)	(*n* = 174)	(*n* = 166)	(*n* = 156)	(*n* = 148)	
**Iron (μg/L)**	SCM	601 ± 392 *	461 ± 310*	346 ± 162	292 ± 141	264 ± 140	216 ± 103	<0.0001
		(*n* = 91)	(*n* = 97)	(*n* = 84)	(*n* = 78)	(*n* = 74)	(*n* = 71)	
	Normal	415 ± 193	389 ± 186	326 ± 137	278 ± 132	245 ± 127	262 ± 502	
		(*n* = 111)	(*n* = 192)	(*n* = 174)	(*n* = 166)	(*n* = 156)	(*n* = 148)	
**Selenium (μg/L)**	SCM	37 ± 19 *	19 ± 6	15 ± 3	12 ± 3	10 ± 2	9 ± 2	<0.0001
		(*n* = 91)	(*n* = 97)	(*n* = 84)	(*n* = 78)	(*n* = 74)	(*n* = 71)	
	Normal	26 ± 12	18 ± 3	15 ± 3	12 ± 2	10 ± 2	10 ± 6	
		(*n* = 111)	(*n* = 192)	(*n* = 174)	(*n* = 166)	(*n* = 156)	(*n* = 148)	
**Manganese (μg/L)**	SCM	6.6 ± 3.9 *	4.4 ± 1.6	3.7 ± 1.1	4.5 ± 3.4	3.4 ± 0.8	3.7 ± 0.9	<0.0001
		(*n* = 79)	(*n* = 41)	(*n* = 26)	(*n* = 22)	(*n* = 19)	(*n* = 13)	
	Normal	5.8 ± 3.1	3.9 ± 1.3	3.5 ± 1.0	3.4 ± 0.9	3.3 ± 0.7	4.0 ± 2.2	
		(*n* = 92)	(*n* = 103)	(*n* = 75)	(*n* = 45)	(*n* = 36)	(*n* = 37)	
**Zinc (μg/L)**	*SCM*	7975 ± 3093 *	3234 ± 1274	2422 ± 906	1616 ± 1208	1113 ± 521	996 ± 520	<0.0001
		(*n* = 91)	(*n* = 97)	(*n* = 84)	(*n* = 78)	(*n* = 74)	(*n* = 71)	
	*Normal*	7178 ± 2734	3590 ± 1051	2686 ± 872	1599 ± 692	1219 ± 571	1031 ± 563	
		(*n* = 111)	(*n* = 192)	(*n* = 174)	(*n* = 166)	(*n* = 153)	(*n* = 148)	
**Copper (μg/L)**	SCM	533 ± 267 *	529 ± 135	423 ± 86	325 ± 74	268 ± 72	221 ± 69	<0.001
		(n = 91)	(n = 97)	(n = 84)	(n = 78)	(n = 74)	(n = 71)	
	Normal	472 ± 175	522 ± 111	422 ± 89	304 ± 76	256 ± 77	228 ± 89	
		(*n* = 111)	(*n* = 192)	(*n* = 174)	(*n* = 166)	(*n* = 156)	(*n* = 148)	

^1^ The mineral and trace element concentrations of HM across lactation were analyzed by using linear mixed model with milk concentration (of a particular mineral and trace element) as dependent variables with the age of infant, SCM status, interaction between the age and the SCM status, the country of origin, the mode of delivery, and the gestational age of the infant at birth as factors. * Statistical significance of the difference between mothers with SCM and without SCM for a particular time point with *p* value <0.05.

**Table 4:** Pattern of infants born to SCM and normal mothers at each time point.

Days Postpartum		Feeding Frequency (per day)		Intake Per Feed (kg/feed)		Daily intake (kg/day)	
		Median	Average	Median	Average	Median	Average
		(Q1, Q3)	(± SD)	(Q1, Q3)	(± SD)	(Q1, Q3)	(± SD)
Day 2	SCM	9 (8, 10.75)	9.56 ± 2.25	0.02 (0.01, 0.04)	0.03 ± 0.03	0.21 (0.12, 0.36)	0.29 ± 0.22
	Normal	9 (8, 11)	9.54 ± 2.55	0.03 (0.02, 0.04)	0.03 ± 0.02	0.27 (0.18, 0.35)	0.28 ± 0.15
Day 17	SCM	8 (6.75, 8)	7.80 ± 1.51	0.07 (0.04, 0.10)	0.07 ± 0.04	0.52 (0.36, 0.74)	0.55 ± 0.24
	Normal	9 (7, 10)	8.9 ± 1.95	0.07 (0.05, 0.09)	0.07 ± 0.04	0.60 (0.45, 0.70)	0.61 ± 0.20
Day 30	SCM	7.5 (7, 9)	7.94 ± 1.95	0.09 (0.06, 0.11)	0.09 ± 0.04	0.66 (0.57, 0.73)	0.67 ± 0.18
	Normal	8 (7, 10)	8.39 ± 1.88	0.09 (0.06, 0.12)	0.09 ± 0.04	0.70 (0.54, 0.87)	0.73 ± 0.25
Day 60	SCM	7 (6,8)	7.24 ± 1.64	0.10 (0.06, 0.13)	0.10 ± 0.04	0.72 (0.61, 0.82)	0.69 ± 0.18
	Normal	7 (6, 8)	7.13 ± 1.78	0.11 (0.08, 0.14)	0.11 ± 0.05	0.75 (0.60, 0.90)	0.77 ± 0.27
Day 90	SCM	7 (6, 8.25)	7.33 ± 1.78	0.12 (0.10, 0.14)	0.12 ± 0.03	0.94 * (0.68, 1.03)	0.89 * ± 0.25
	Normal	6 (5, 7)	6.26 ± 1.58	0.12 (0.09, 0.16)	0.13 ± 0.05	0.75 (0.62, 0.90)	0.76 ± 0.21
Day 120	SCM	6 (6, 7.5)	6.47 ± 1.36	0.13 (0.08, 0.15)	0.12 ± 0.05	0.75 (0.66, 0.81)	0.72 ± 0.25
	Normal	6 (5, 7)	5.94 ± 1.62	0.13 (0.09, 0.17)	0.14 ± 0.06	0.73 (0.66, 0.91)	0.77 ± 0.23

* *p* = 0.032.

**Table 5:** Growth parameters of infants of SCM and normal mothers.

	V0 (Child Birth)		Overall Effect	
Z Score	Estimate SCM vs. Normal (SE)	*p* Value	Estimate SCM vs. Normal (SE) ^1^	*p* Value ^1^
BMI	−0.477 (0.134)	0.0005	0.032 (0.066)	0.624
Head circumference	−0.581 (0.135)	<0.0001	−0.022 (0.056)	0.696
Length	−0.251 (0.130)	0.059	−0.073 (0.053)	0.165
Weight	−0.430 (0.121)	0.0005	0.011 (0.049)	0.831
Weight for length	−0.421 (0.142)	0.004	0.056 (0.073)	0.448

^1^ The infant’s growth Z-scores were analyzed by using linear mixed model with Z-score of different growth parameter as dependent variable with the age of infant, SCM status, interaction between age and the SCM status, the baseline value of the corresponding growth parameter and the gestational age of the infant at birth as explanatory variable.

## References

[1] Aryeetey R.N.O., Marquis G.S., Timms L., Lartey A., Brakohiapa L. (2008). Subclinical mastitis is common among ghanaian women lactating 3 to 4 months postpartum. J. Hum. Lact..

[2] Morton J.A. (1994). The Clinical Usefulness of Breast Milk Sodium in the Assessment of Lactogenesis. Pediatrics.

[3] Gomo E., Filteau S.M., Tomkins A.M., Ndhlovu P., Michaelsen K.F., Friis H. (2003). Subclinical mastitis among HIV-infected and uninfected Zimbabwean women participating in a multimicronutrient supplementation trial. Trans. R. Soc. Trop. Med. Hyg..

[4] Arsenault J.E., Aboud S., Manji K.P., Fawzi W.W., Villamor E. (2010). Vitamin Supplementation Increases Risk of Subclinical Mastitis in HIV-Infected Women. J. Nutr..

[5] Nussenblatt V., Lema V., Kumwenda N., Broadhead R., Neville M.C., Taha T.E., Semba R.D. (2005). Epidemiology and microbiology of subclinical mastitis among HIV-infected women in Malawi. Int. J. STD AIDS.

[6] Neville M.C., Allen J.C., Archer P.C., Casey C.E., Seacat J., Keller R.P., Lutes V., Rasbach J., Neifert M. (1991). Studies in human lactation: Milk volume and nutrient composition during weaning and lactogenesis. Am. J. Clin. Nutr..

[7] Thomsen A.C., Hansen K.B., Moller B.R. (1983). Leukocyte counts and microbiologic cultivation in the diagnosis of puerperal mastitis. Am. J. Obstet. Gynecol..

[8] Filteau S.M., Lietz G., Mulokozi G., Bilotta S., Henry C.J.K., Tomkins A.M. (1999). Milk cytokines and subclinical breast inflammation in Tanzanian women: Effects of dietary red palm oil or sunflower oil supplementation. Immunology.

[9] World Health Organization (2000). Mastitis-Causes and Management.

[10] Michie C., Lockie F., Lynn W. (1984). The Challenge of Addiction. Lancet.

[11] Fetherston C. (1998). Risk Factors for Lactation Mastitis. J. Hum. Lact..

[12] Willumsen J.F., Filteau S.M., Coutsoudis A., Uebel K.E., Newell M.-L., Tomkins A.M. (2000). Subclinical Mastitis as a Risk Factor for Mother-Infant HIV Transmission. Adv. Exp. Med. Biol..

[13] Kantarci S., Koulinska I.N., Aboud S., Fawzi W.W., Villamor E. (2007). Subclinical mastitis, cell-associated HIV-1 shedding in breast milk, and breast-feeding transmission of HIV-1. J. Acquir. Immune Defic. Syndr..

[14] Batavani R.A., Asri S., Naebzadeh H. (2007). The effect of subclinical mastitis on milk composition in dairy cows. Iran. J. Vet. Res..

[15] Bruckmaier R.M., Ontsouka C.E., Blum J.W. (2004). Fractionized milk composition in dairy cows with subclinical mastitis. Vet. Med. Czech.

[16] Schukken Y.H., Günther J., Fitzpatrick J., Fontaine M.C., Goetze L., Holst O., Leigh J., Petzl W., Schuberth H., Sipka A. (2011). Host-response patterns of intramammary infections in dairy cows. Vet. Immunol. Immunopathol..

[17] Li C., Solomons N.W., Scott M.E., Koski K.G. (2018). Subclinical mastitis (SCM) and proinflammatory cytokines are associated with mineral and trace element concentrations in human breast milk. J. Trace Elem. Med. Biol..

[18] Leitner G., Chaffer M., Shamay A., Shapiro F., Merin U., Ezra E., Saran A., Silanikove N. (2004). Changes in milk composition as affected by subclinical mastitis in sheep. J. Dairy Sci..

[19] Busato A., Trachsel P., Schällibaum M., Blum J.W. (2000). Udder health and risk factors for subclinical mastitis in organic dairy farms in Switzerland. Prev. Vet. Med..

[20] Schrick F.N., Hockett M.E., Saxton A.M., Lewis M.J., Dowlen H.H., Oliver S.P. (2001). Influence of Subclinical Mastitis During Early Lactation on Reproductive Parameters. J. Dairy Sci..

[21] Singh M., Yadav P., Sharma A., Garg V.K., Mittal D. (2017). Estimation of Mineral and Trace Element Profile in Bubaline Milk Affected with Subclinical Mastitis. Biol. Trace Elem. Res..

[22] Silanikove N., Merin U., Leitner G. (2014). On effects of subclinical mastitis and stage of lactation on milk quality in goats. Small Rumin. Res..

[23] Tuaillon E., Viljoen J., Dujols P., Cambonie G., Rubbo P.A., Nagot N., Bland R.M., Badiou S., Newell M.L., Van De Perre P. (2017). Subclinical mastitis occurs frequently in association with dramatic changes in inflammatory/anti-inflammatory breast milk components. Pediatr. Res..

[24] Aryeetey R.N.O., Marquis G.S., Brakohiapa L., Timms L., Lartey A. (2009). Subclinical Mastitis May Not Reduce Breastmilk Intake During Established Lactation. Breastfeed. Med..

[25] Kasonka L., Makasa M., Marshall T., Chisenga M., Sinkala M., Chintu C., Kaseba C., Kasolo F., Gitau R., Tomkins A. (2006). Risk factors for subclinical mastitis among HIV-infected and uninfected women in Lusaka, Zambia. Paediatr. Perinat. Epidemiol..

[26] Cruz-hernandez C., Thakkar S.K., Masserey-elmelegy I., Bousi W., Fontannaz P., Giuffrida F. (2017). Quantification of Fatty Acids in Erythrocytes and Plasma by Fast Gas Chromatography. J. Sep. Sci..

[27] Affolter M., Garcia-Rodenas C.L., Vinyes-Pares G., Jenni R., Roggero I., Avanti-Nigro O., de Castro C.A., Zhao A., Zhang Y., Wang P. (2016). Temporal changes of protein composition in breast milk of Chinese urban mothers and impact of caesarean section delivery. Nutrients.

[28] Klein P.D., Butte N.F., Garza C., Patterson B.W., Wong W.W. (2018). Human-milk intake measured by administration of deuterium oxide to the mother: A comparison with the test-weighing technique. Am. J. Clin. Nutr..

[29] Macfarlane A.J., Blondel B., Mohangoo A.D., Cuttini M., Nijhuis J., Novak Z., Ólafsdóttir H.S., Zeitlin J. (2016). Wide differences in mode of delivery within Europe: Risk-stratified analyses of aggregated routine data from the Euro-Peristat study. BJOG.

[30] Khanal V., Scott J.A., Lee A.H., Binns C.W. (2015). Incidence of Mastitis in the Neonatal Period in a Traditional Breastfeeding Society: Results of a Cohort Study. Breastfeed. Med..

[31] Say B., Dizdar E.A., Degirmencioglu H., Uras N., Sari F.N., Oguz S., Canpolat F.E. (2016). The effect of lactational mastitis on the macronutrient content of breast milk. Early Hum. Dev..

[32] Hunt K.M., Williams J.E., Shafii B., Hunt M.K., Behre R., Ting R., McGuire M.K., McGuire M.A. (2013). Mastitis Is Associated with Increased Free Fatty Acids, Somatic Cell Count, and Interleukin-8 Concentrations in Human Milk. Breastfeed. Med..

[33] Prentice A., Prentice A.M., Lamb W.H. (1985). Mastitis in rural Gambian mothers and the protection of the breast by milk antimicrobial factors. Trans. R. Soc. Trop. Med. Hyg..

[34] Fetherston C.M., Lai C.T., Hartmann P.E. (2006). Relationships Between Symptoms and Changes in Breast Physiology During Lactation Mastitis. Breastfeed. Med..

[35] Ramadan M.A., Salah M.M., Eid S.Z. (1972). The effect of breast infection on the composition of human milk. Int. J. Biochem..

[36] Ogola H., Shitandi A., Nanua J. (2007). Effect of mastitis on raw milk compositional quality. J. Vet. Sci..

[37] Auldist M.J., Coats S., Rogers G.L., McDowell G.H. (1995). Changes in the Composition of Milk From Healthy and Mastitic Dairy-Cows During the Lactation Cycle. Aust. J. Exp. Agric..

[38] Buescher E.S., Hair P.S. (2001). Human milk anti-inflammatory component contents during acute mastitis. Cell. Immunol..

[39] Layman D.K., Lönnerdal B., Fernstrom J.D. (2018). Applications for α-lactalbumin in human nutrition. Nutr. Rev..

[40] Kasuga K., Yang R., Porter T.F., Agrawal N., Petasis N.A., Irimia D., Toner M., Serhan C.N. (2008). Rapid Appearance of Resolvin Precursors in Inflammatory Exudates: Novel Mechanisms in Resolution. J. Immunol..

[41] Hogan J.S., Weiss W.P., Smith K.L. (1993). Role of Vitamin E and Selenium in Host Defense Against Mastitis. J. Dairy Sci..

[42] Smith K.L., Harrison J.H., Hancock D.D., Todhunter D.A., Conrad H.R. (1984). Effect of Vitamin E and Selenium Supplementation on Incidence of Clinical Mastitis and Duration of Clinical Symptoms. J. Dairy Sci..

[43] Smith K.L., Hogan J.S., Weiss W.P. (1997). Dietary vitamin E and selenium affect mastitis and milk quality. J. Anim. Sci..

[44] Duntas L.H. (2009). Selenium and inflammation: Underlying anti-inflammatory mechanisms. Horm. Metab. Res..

[45] Overton T.R., Yasui T. (2014). Practical applications of trace minerals for dairy cattle. J. Anim. Sci..

[46] Scaletti R.W., Trammell D.S., Smith B.A., Harmon R.J. (2003). Role of Dietary Copper in Enhancing Resistance to Escherichia coli Mastitis. J. Dairy Sci..

[47] Wegner T.N., Stull J.W. (1978). Relation between mastitis test score, mineral composition of milk, and blood electrolyte profiles in Holstein cows. J. Dairy Sci..

[48] Martinez N., Sinedino L.D.P., Bisinotto R.S., Ribeiro E.S., Gomes G.C., Lima F.S., Greco L.F., Risco C.A., Galvão K.N., Taylor-Rodriguez D. (2014). Effect of induced subclinical hypocalcemia on physiological responses and neutrophil function in dairy cows. J. Dairy Sci..

[49] Scanlon K.S., Alexander M.P., Serdula M.K., Davis M.K., Bowman B.A. (2002). Assessment of Infant Feeding: The Validity of Measuring Milk Intake. Nutr. Rev..

[50] Savenije O.E., Brand P.L. (2006). Accuracy and precision of test weighing to assess milk intake in newborn infants. Arch. Dis. Child. Fetal Neonatal Ed..

[51] Drewett R.F., Woolridge M.W., Greasley V., Mcleod C.N., Hewison J., Williams A.F., Baum J.D. (1984). Evaluating breast-milk intake by test weighing: A portable electronic balance suitable for community and field studies. Early Hum. Dev..

[52] Manganaro R., Marseglia L., Mamì C., Palmara A., Paolata A., Loddo S., Gargano R., Mondello M., Gemelli M. (2007). Breast milk sodium concentration, sodium intake and weight loss in breast-feeding newborn infants. Br. J. Nutr..

[53] Wren-Atilola H.M., Solomons N.W., Scott M.E., Koski K.G. (2019). Infant growth faltering linked to subclinical mastitis, maternal faecal–oral contamination, and breastfeeding. Matern. Child Nutr..

[54] Filteau S.M., Rice A.L., Ball J.J., Chakraborty J., Stoltzfus R., De Francisco A., Willumsen J.F. (1999). Breast milk immune factors in Bangladeshi women supplemented postpartum with retinol or β-carotene. Am. J. Clin. Nutr..

[55] Li C., Solomons N.W., Scott M.E., Koski K.G. (2019). Anthropometry before Day 46 and Growth Velocity before 6 Months of Guatemalan Breastfed Infants Are Associated with Subclinical Mastitis and Milk Cytokines, Minerals, and Trace Elements. J. Nutr..

